# Comparison of implant placement accuracy in two different preoperative digital workflows: navigated vs. pilot-drill-guided surgery

**DOI:** 10.1186/s40729-021-00322-1

**Published:** 2021-04-30

**Authors:** Johannes Spille, Feilu Jin, Eleonore Behrens, Yahya Açil, Jürgen Lichtenstein, Hendrik Naujokat, Aydin Gülses, Christian Flörke, Jörg Wiltfang

**Affiliations:** 1 Christian Albrechts University, Department of Oral and Maxillofacial Surgery, University Hospital of Schleswig-Holstein, Campus Kiel, Arnold-Heller-Straße 3, 24105 Kiel, Germany; 2 Department of oral and Maxillofacial Surgery, School of Medicine, 2nd Affiliated Hospital of Zhejiang University, Hangzhou, Zhejiang China

**Keywords:** Navigation, Accuracy, Implant system, Digital workflow

## Abstract

**Background:**

The aim of the study is to evaluate the accuracy of a new implant navigation system on two different digital workflows.

**Methods:**

A total of 18 phantom jaws consisting of hard and non-warping plastic and resembling edentulous jaws were used to stimulate a clinical circumstance. A conventional pilot-drill guide was conducted by a technician, and a master model was set by using this laboratory-produced guide. After cone beam computed tomography (CBCT) and 3D scanning of the master models, two different digital workflows (marker tray in CBCT and 3D-printed tray) were performed based on the Digital Imaging Communication in Medicine files and standard tessellation language files. Eight Straumann implants (4.1 mm × 10 mm) were placed in each model, six models for each group, resulting in 144 implant placements in total. Postoperative CBCT were taken, and deviations at the entry point and apex as well as angular deviations were measured compared to the master model.

**Results:**

The mean total deviations at the implant entry point for MTC (marker tray in CBCT), 3dPT (3d-printed tray), and PDG (pilot-drill guide) were 1.024 ± 0.446 mm, 1.027 ± 0.455 mm, and 1.009 ± 0.415 mm, respectively, and the mean total deviations at the implant apex were 1.026 ± 0.383 mm, 1.116 ± 0.530 mm, and 1.068 ± 0.384 mm. The angular deviation for the MTC group was 2.22 ± 1.54°. The 3dPT group revealed an angular deviation of 1.95 ± 1.35°, whereas the PDG group showed a mean angular deviation of 2.67 ± 1.58°. Although there were no significant differences among the three groups (*P* > 0.05), the navigation groups showed lesser angular deviations compared to the pilot-drill-guide (PDG) group. Implants in the 3D-printed tray navigation group showed higher deviations at both entry point and apex.

**Conclusions:**

The accuracy of the evaluated navigation system was similar with the accuracy of a pilot-drill guide. Accuracy of both preoperative workflows (marker tray in CBCT or 3D-printed tray) was reliable for clinical use.

## Background

Precise placement of implants is of great importance in survival of an implant-supported prosthesis [1]. Implant malposition was reported to be the most likely reason to cause peri-implantitis. It has been proclaimed that approximately 50% of all peri-implantitis cases had been triggered by implant malposition during surgery [2]. To achieve the optimal functional and esthetic results, the concept of “guided surgery” was introduced to allow clinicians to achieve higher precision of dental implant placement [3, 4]. Nowadays, a guided surgery could be performed by using either surgical guides or dynamic navigation systems. Although computer-aided implant systems have been proved to improve the accuracy in either in vivo or in vitro studies [5, 6], a conventional surgical template is still the first choice to many surgeons, considering the cost and complexity of computer-aided guidance [7].

A conventional surgical guide is known to be fabricated on a gypsum cast by the technician, which is still the most frequently used in clinical circumstances, followed by computer-aided design/computer-aided manufacturing (CAD/CAM) static guidance [8].

Although multiple studies have proved the use of surgical guides could improve the accuracy of final implant position [9, 10], certain limitations should still be considered such as poor visualization of anatomical structures, poor bone cooling during osteotomy, a possibility of loosening the template during drilling, and problems related to the intraoperative modification of the preoperative plan [11–13].

In recent years, dynamic navigation systems were developed to find a better solution to solve these problems. Supported by a computed-navigation system, pre-planned position of implant and real-time position of the drill tip could be easily detected on a screen [12]. Therefore, suitable adjustment can be made and a more precise placement could be ensured [13].

A digital plan based on preoperatively marked cone beam computed tomography (CBCT) images is a widely accepted planning modality by both clinicians and researchers. However, an intraoral scanning to gain the standard tessellation language (STL) files followed by a 3D-printed tray seems to be also promising [14].

The aim of the current study was to assess the accuracy of implant placement using a new navigation system in comparison with CBCT (MTC) and 3D-printed tray (3dPT) workflows.

## Material and methods

### Study design

On Frasaco models, which have exhibited the same clinical situation, the following three insertion protocols were established:

- Pilot-drill guided
- Marker tray in CBCT navigation
- 3D-printed tray navigation

A single researcher has planned the locations of the implants in the jaw prior to experiments. In order to avoid possible bias, all implant insertions were performed by another single, blinded researcher for all groups, who was not involved in the evaluation process of the accuracy. Another researcher, who was blinded to the preoperative data plan, has conducted the postoperative DVT scans and determined the positions of the inserted implants compared to the master typodont. The calculation of the accuracy values was then carried out automatically using selected computer programs. All results were included in this study; no results were discarded or recalculated.

The study evaluates the accuracy of implant placement in Frasaco models under instruction from the Denacam System of the company mininavident AG (Liestal, Schweiz). Denacam is a dynamic computer-assisted surgical system and uses the principles of stereo triangulation from optical cameras. As a real-time navigation system, Denacam uses a small, prefabricated intraoral marker to coordinate the planned implant position and the real-time position of the drill during the operation. The practician recognizes during the operation deviations in the entry point, the apex, and the angle on a screen. In this way, the current drill position and the planned implant insertion can be coordinated.

### Fabrication of the surgical guide

**Fig. 1:**
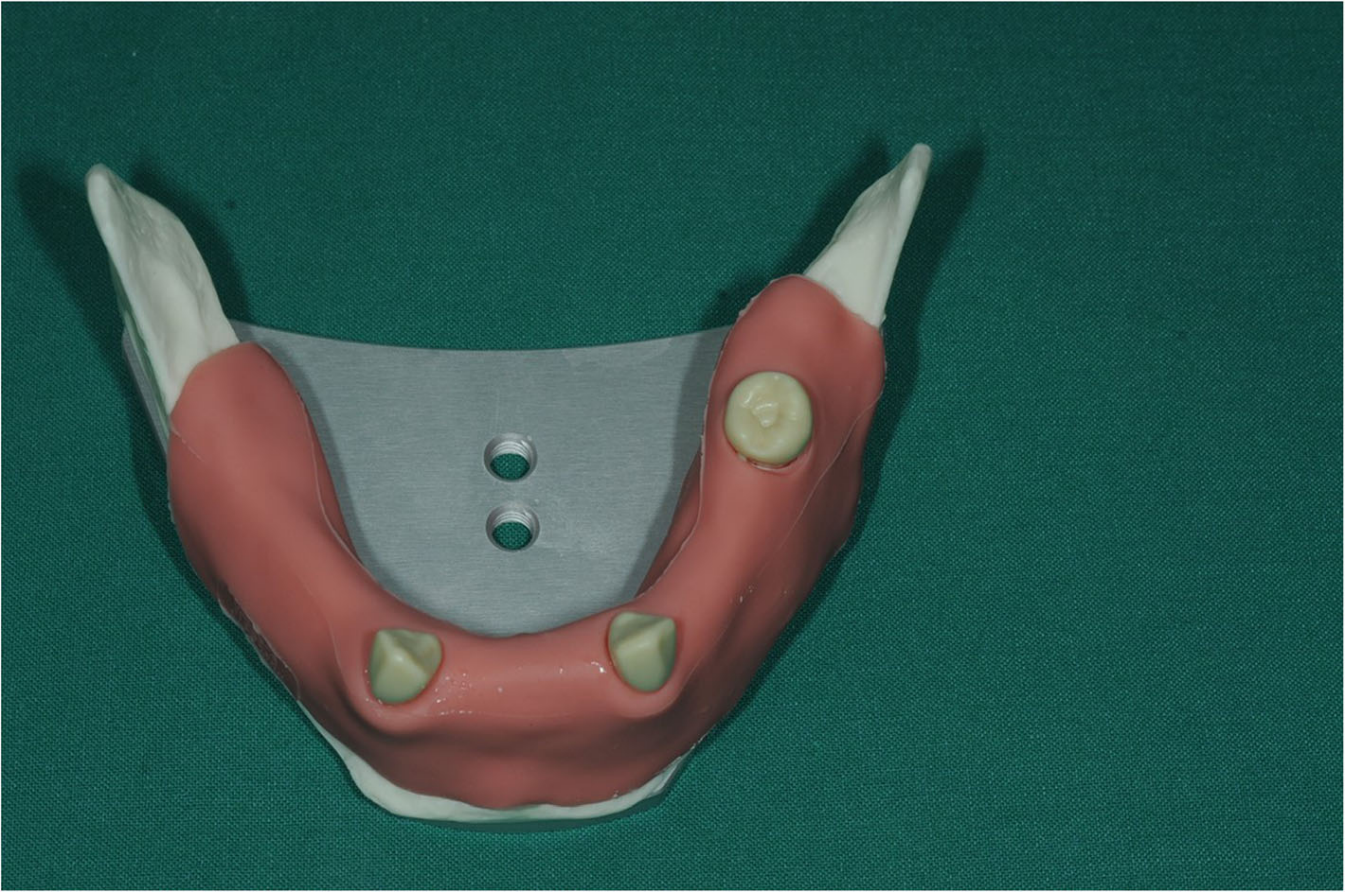
Original Frasaco mandible

**Fig. 2:**
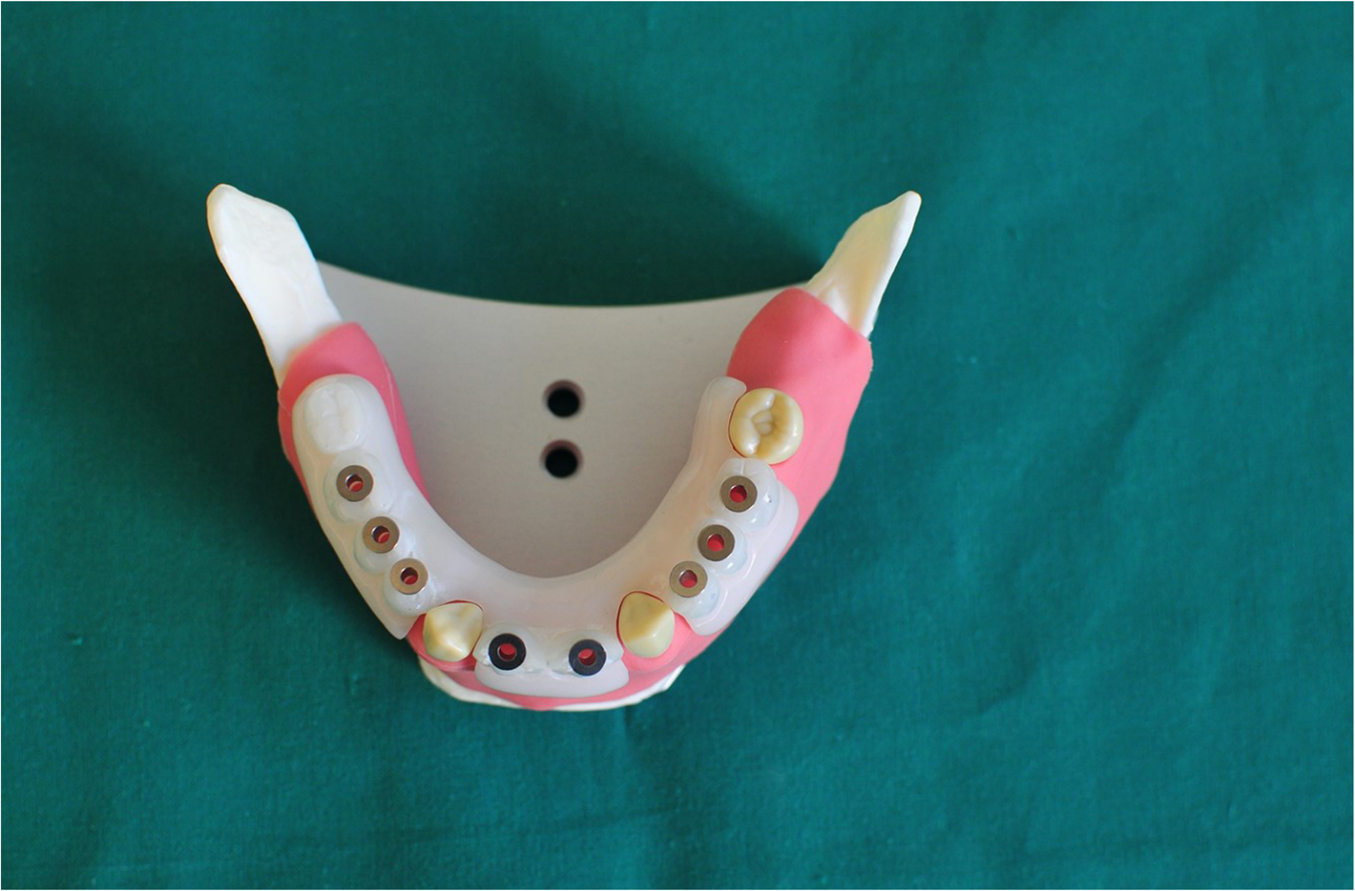
Frasaco jaw with pilot-drill guide on

Partially edentulous Frasaco mandible (Frasaco GmbH, Tettnang, Germany) was used as the master typodont (Fig. 1). Eleven teeth on the jaw were missing in total, and four teeth on each side were decided to be replaced with implants, which made eight implants in total. A wax-up prothesis was firstly accomplished to mimic an ideal denture on a duplicated cast model. Then, the final acrylic-made pilot-drill guide was fabricated based on the wax prosthesis (Fig. 2). Eight Steco titanium drill sleeves (2.2 mm inner, 3.5 mm outer, and 5.0 mm in length; Steco-system-technik GmbH&Co.KG) corresponding to planned implant positions were inserted into the guide to guarantee a more stable guidance.

### Preparation of the master typodont

The drillings on the master typodont were firstly guided by the pilot-drill guide, and as for the twist drills, parallel pins in neighboring drill holes were used to maintain proper angles. Eight Straumann bone level implants (4.1 mm × 10 mm; Straumann Holding AG, Switzerland) were placed manually into the implant beds after osteotomies.

### Workflow for the marker tray in CBCT navigation (MTCN) group

**Fig. 3:**
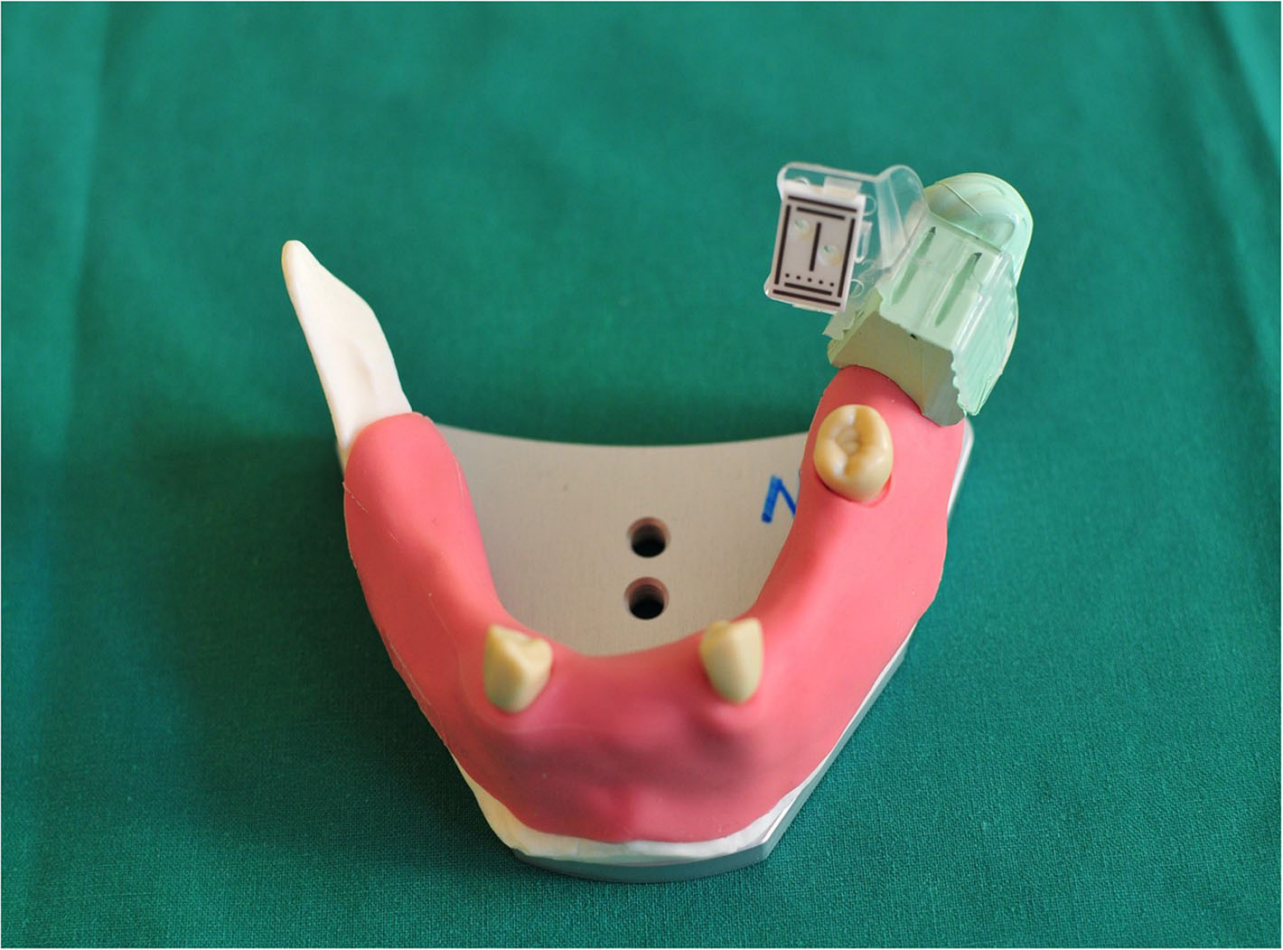
Frasaco jaw with marker tray on

To register the jaw into the navigation system, a marker tray with the registration marker on was firmly adapted to the master typodont’s left ramus by using vinylpolysiloxane impression material (Flexitime, KULZER GmbH) (Fig. 3). Usually, this type of system has a registration marker, which should be fixed on the remaining teeth at the same dental arch during the implant insertion. In the current study, the marker tray was placed on the left ascending ramus; thus, a total of eight implants should be placed. The re-positioning of the marker could negatively influence the accuracy of the measurement. The master typodont was then scanned by CBCT machine (KaVo 3D eXam, resolution: 0.2 voxel), and the Digital Imaging Communication in Medicine (DICOM) files were imported into the coDiagnostiX system (Dental wings GmbH, Chemnitz, Germany). The geometry of the implants was easily recognized, and the virtual implants were located more precisely by superimposing upon the images. The digital plan was imported into the navigation system afterwards. The slice thickness of the CBCT machine was set to 0.2 voxel on application of Denacam System, which had no significant effect on the clinical application.

### Workflow for the 3D-printed tray navigation (3dPTN) group

**Fig. 4:**
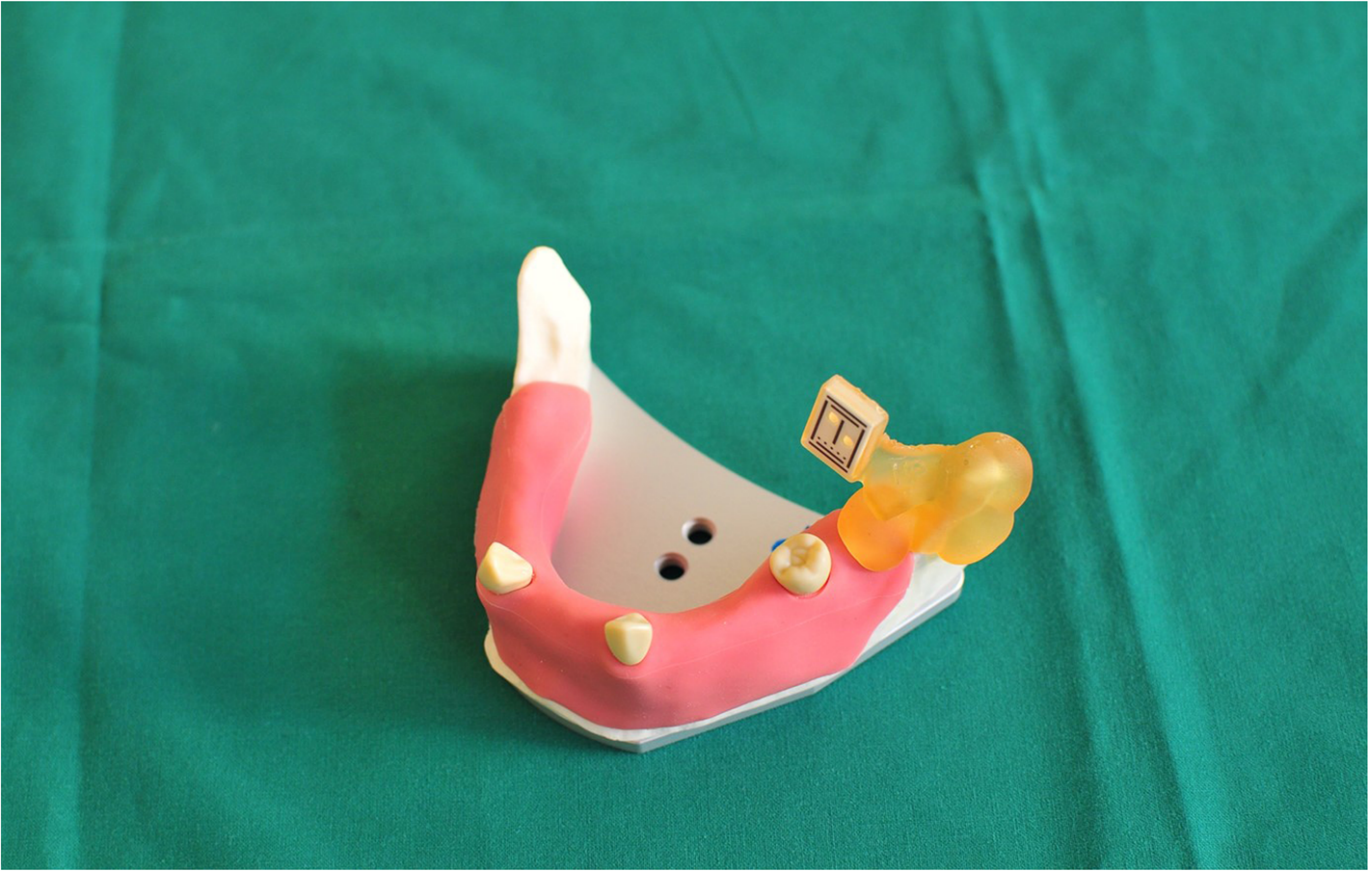
Frasaco jaw with 3D-printed maker tray on

Both the master typodont with original marker tray on and the particular Frasaco typodont which was chosen for the 3dPTN group were scanned by Shining 3D (Shining 3D Tech. Co. Ltd. Hangzhou, China). The STL files obtained were imported into coDiagnostiX, and three match regions on both typodonts were chosen to do the superimposition, thus, exact position of the marker on master typodont was copied to the 3dPTN model. The STL model of the tray and the marker provided by the same company as the navigation system was superimposed onto the original tray. New STL files of the 3dPTN model with the virtual marker tray in the same position as the master typodont were created. The new STL files were then imported to a 3D-printing software, and the newly designed tray was printed in resin by a Form2 printer (Formlabs Boston Experience, USA, thickness of layer: 0.1 mm) (Fig. 4).

### Surgical protocol

**Fig. 5:**
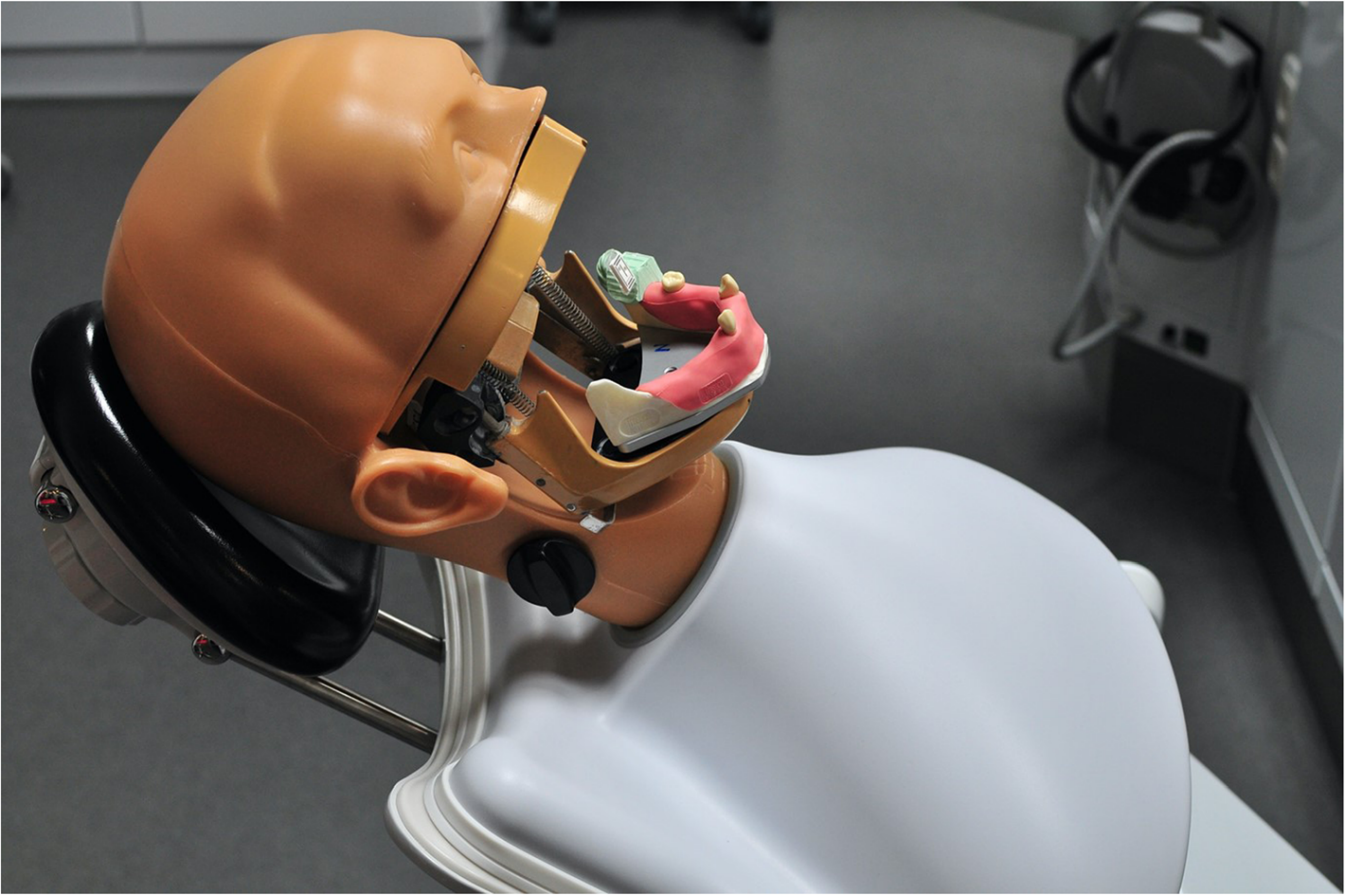
Frasaco jaw on a phantom head

**Fig. 6:**
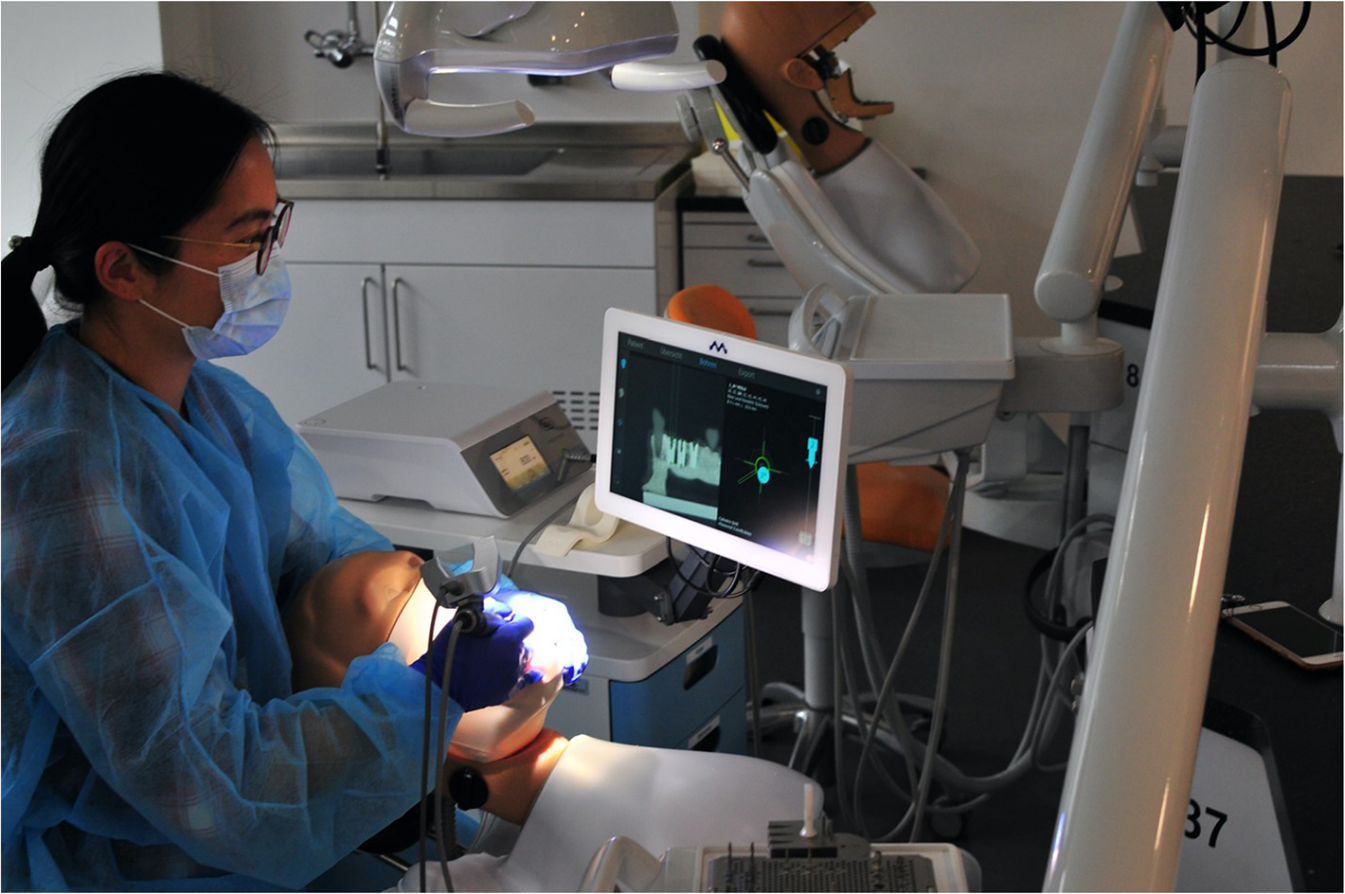
During the operation

The typodont was fixed in a Frasaco phantom head before surgery (Figs. 5 and 6). The soft silicone tissue was then prepared, and the alveolar ridge was exposed. Both the laboratory guide group and the navigation groups followed the standard Straumann drill protocol: a 1.4-mm round burr was set to define the entry point firstly. A 2.2-mm pilot drill followed by 2.8-mm and 3.5-mm twist drills, except for the navigation groups, was used. Eight Straumann implants with the same size as the master typodont were placed manually in each typodont.

Postoperative CBCT of each typodont was taken, and the bore holes were refilled with a special material (A-J OP UK K, Frasaco GmbH, Tettnang, Germany). The re-cemented typodonts were then used for the next trial. Six trials for each group were performed, and DICOM files were obtained for further measurements.

### Pilot-drill guided group

The surgical guide was seated properly during the whole drilling procedure. After the pilot-drill drilling, the surgical guide was removed, and the twist drills were guided by parallel pins. The laser marker on the drill was used to guide the depth.

### Marker tray in CBCT navigation group

The same registration tray used for preoperative CBCT scan was seated firmly on Frasaco ramus, and each burr used was registered before drilling. If the camera was rotated for a better detection of the marker, the burr should be registered again afterwards.

### 3D-printed tray navigation group

The printed marker tray was seated on the left ramus as well. The same registration procedure and drilling protocol was followed.

### Measurements of error

**Fig. 7:**
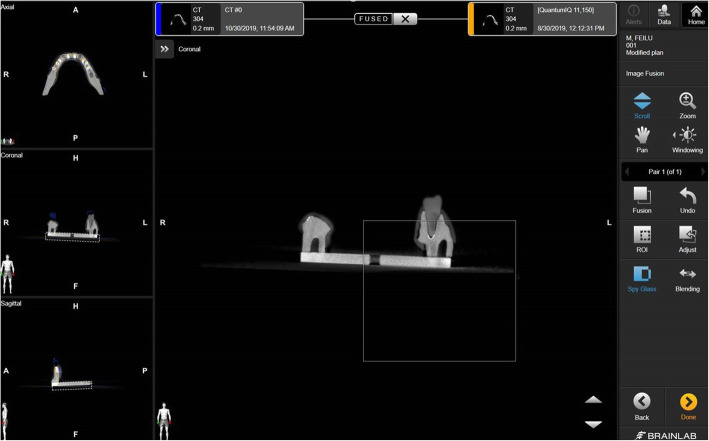
CBCT images of test model and master model were fused based on the metal base

Postoperative DICOM files of master typodont as well as one experimental typodont were imported into the Brainlab planning software (version 3.1; BrainlabAG, Munich, Germany). The rigid metal base of the typodont was used to do image fusion, and a spy glass function was used to check (Fig. 7). Once the images were fused approvingly, a linked view would be shown in double windows format.

The entry point at implant shoulder and the apex of each implant were defined on a sagittal view first, and a virtual axial line was drawn. Distances between entry points or apexes were considered to be total error, and deviations on axial view were defined as lateral errors which represented a mesial-distal deviation and buccal-lingual deviation combined. Error of depth was calculated using the square root formula. The open angle function was used to measure angular error between two virtual axial lines.

### Statistical analysis

SPSS software package (IBM SPSS Statics, version 26.0, IBM) was used, and homogeneity of each parameter among the three groups was checked firstly by means of Leven’s test. All the seven parameters turned out to be homogenous. Then, the one-way analysis of variance (ANOVA) using Scheffe’s test as a post-hoc analysis was carried out to analyze the differences among the three groups. *P* value < 0.05 was considered to be statistically significant.

## Results

**Fig. 8:**
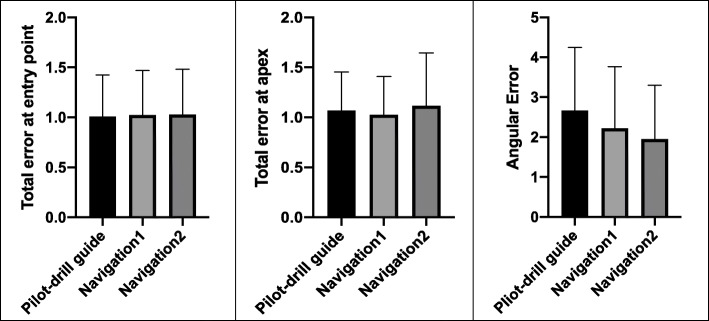
Comparison of the three groups “pilot-drill guided,” “marker tray in CBCT,” and “3D-printed tray navigation” regarding the total error at entry point, the total error at apex, and the angular error

A total of 144 implants (*n* = 48 for each group) were inserted. The mean values and standard deviations of each parameter were established in Table 1. Although there were no significant differences among the three groups (*P* > 0.05), the navigation groups showed lower angular deviations compared to the pilot-drill guide (PDG) group (2.67 ± 1.58°), which were 1.95 ± 1.35° for the 3dPTN group and 2.22 ± 1.54° for the MTCN group, respectively. Besides, the 3dPTN group showed the greatest deviations at both entry point and apex. The PDG group established the best result of total error at entry point, which was 1.009 ± 0.415 mm, followed by 1.024 ± 0.446 mm for the MTCN group and 1.027 ± 0.455 mm for the 3dPTN group, respectively (Fig. 8).

**Table 1:** Mean values and standard deviations of each parameter

Groups	Total error at entry point (mean ± SD)	Total error at apex (mean ± SD)	Angular error (mean ± SD)
Pilot-drill guide	1.009 ± 0.415 mm	1.068 ± 0.384 mm	2.67 ± 1.58°
Navigation1 (marker tray in CBCT)	1.024 ± 0.446 mm	1.026 ± 0.383 mm	2.22 ± 1.54°
Navigation2 (3D-printed tray)	1.027 ± 0.455 mm	1.116 ± 0.530 mm	1.95 ± 1.35°

## Discussion

In this study, attachment of the optical camera put extraweight on the handpiece, which would make the operator lose dexterity when firstly use the equipment. That might be the reason why errors at entry point for the navigation groups were greater than the PDG group. Moreover, angular deviations for the navigation groups were lower than the PDG group, which could be attributed to the modification of the drilling angles and depth based on the real-time feedback on the screen. As for the PDG group, depth control depended on the laser marker on the drill and angular adjustment was less likely to happen once the surgeon started drilling. Chen et al. (2018) reported similar results using a different navigation system on models; total error at entry point was 1.07 ± 0.48 mm, total error at apex was 1.35 ± 0.55 mm, and angular error was 4.45 ± 1.97° [15]. Jorba-Garcia et al. (2019) and Sun et al. (2018) also reported approximate results [16, 17]. With regard to limited studies of navigation systems, an accuracy of 1–2 mm in vitro has been reported generally [18]. Besides that, discrepancies were usually smaller in in vitro studies than those in clinical studies, considering the disturbing factors like bad vision, movement of patients, and limited operating time [19]. Dental implant surgery uses global technological advances to enable the best possible dental care [20]. For example, there are first approaches of robotic surgery, which have been proven for both simple and complex clinical cases [21].

Moreover, this study shows that it was sometimes quite difficult to rotate the camera to a proper position to get a whole vision of the marker tray, especially when drilling at the opposite side. Thus, a recognition of this study is that it is better to place the marker tray as close to the osteotomy site as possible to make the process easier.

As for a navigation-guided implant surgery, good hand-eye coordination is of great importance. Operators need to stare at real-time images on the screen instead of the patient’s oral cavity, and even an experienced surgeon can feel less confident at first. Therefore, a learning curve needs to be established. Arora et al. reported that a learning curve of cardio-thoracic and vascular surgical procedures was about 48 studies in total [22]. In this current study, an obvious learning curve was noticed from the first implant to the twenty-fourth implant, so it can be suggested that no less than 25–30 osteotomies should be performed before surgeons treat a real patient with the system.

The most attracting advantage of navigation systems is visualization [23]. Tang et al. (2019) reported that anatomical structures that deviate from the norm can be reasons for greater inaccuracies in free-hand implant placement [24]. Visualization of drill tips makes it possible for real-time correction, and more precise implant position should be promised theoretically. On the other hand, visualization of critical anatomical structures (floor of the sinus, mandibular canal, mental foramen, etc.) reduces the possibility of severe surgical complications. Besides that, navigation systems have more extensive indications compared to surgical guide systems. Navigation-guided implantation could also be more suitable for patients with limited mouth-opening or tight interdental space. As to non-experienced surgeons, the process can be easier with the help of navigation systems.

Despite their advantages in accuracy, it should be kept in mind that the handpiece used by the navigation systems should be occupied by a camera and its weight could influence the learned manipulation of the practitioner. In addition, the marker as an additional medium intraorally could also negatively influence the visual angle, which might lead to inaccuracies [15, 25]. Besides that, the placement of the registration marker by edentulous patients remains still a clinical problem to be solved by the manufacturers.

Further in vitro studies with the Denacam System or equivalent systems must now provide information about the shown data. At least, newer planning software and planning methods enable a postoperative evaluation of the implant positions without having to rely to regular x-rays because of the use of digital casts [26]. The accuracy of the implant placement is a very complex process that is prone to errors. Every single step from scanning, process planning, guide adjustment etc. influences the position of the implants [27]. Digital technologies can increase the accuracy, efficiency, and comfort of implant treatment and achieve satisfactory occlusal reconstruction results in patients with difficult anatomical conditions and complex cases [28, 29].

## Conclusions

The accuracy of the evaluated navigation system was similar as the accuracy of a pilot-drill guide or other reported navigation systems. The accuracy of both preoperative workflows (marker tray in CBCT or 3D-printed tray) was reliable enough for clinical use.

## Data Availability

The datasets used and/or analyzed during the current study are available from the corresponding author on reasonable request.

## Abbreviations

CBCT: Cone beam computed tomography
PDG: Pilot-drill guide
3dPTN: 3D-printed tray navigation
MTCN: Marker tray in CBCT navigation
STL: Standard tessellation language
